# Towards universal chemosensory testing: needs, barriers, and opportunities

**DOI:** 10.1093/chemse/bjaf015

**Published:** 2025-05-20

**Authors:** Steven D Munger, Kai Zhao, Linda A Barlow, Duncan Boak, Katie Boateng, Susan E Coldwell, Pamela Dalton, Richard L Doty, Jennifer E Douglas, Valerie Duffy, Frank A Franklin, John E Hayes, Howard J Hoffman, Patrice Hubert, Paule V Joseph, Jeb M Justice, Joshua M Levy, Julie A Mennella, Marianna Obrist, M Yanina Pepino, Jayant M Pinto, Edmund A Pribitkin, Christopher T Simons, Mark W Albers, Valentina Parma

**Affiliations:** Department of Otolaryngology—Head and Neck Surgery, University of Virginia, Charlottesville, VA 22908, United States; Center for Smell and Taste Disorders, University of Virginia, Charlottesville, VA 22908, United States; Department of Otolaryngology—Head and Neck Surgery, the Ohio State University, 915 Olentangy River Road, Columbus, OH 43212, United States; Department of Cell and Developmental Biology, University of Colorado Anschutz Medical Campus, Aurora, CO 80045, United States; Rocky Mountain Taste and Smell Center, University of Colorado Anschutz Medical Campus, Aurora, CO 80045, United States; Cell Biology, Stem Cells and Development Graduate Program, University of Colorado Anschutz Medical Campus, Aurora, CO 80045, United States; Fifth Sense, Unit 2, Franklins House, Wesley Lane, Bicester OX26 6JU, United Kingdom; The Smell and Taste Association of North America, Philadelphia, PA, United States; Oral Health Sciences, School of Dentistry, University of Washington, Room B-509, Health Sciences Building, Box 357475, Seattle, WA 98195-7475, United States; Monell Chemical Senses Center, 3500 Market Street, Philadelphia, PA, United States; Department of Otorhinolaryngology—Head and Neck Surgery, University of Pennsylvania, Philadelphia, PA 19104, United States; Department of Otorhinolaryngology—Head and Neck Surgery, University of Pennsylvania, Philadelphia, PA 19104, United States; Department of Allied Health Sciences, University of Connecticut, Storrs, CT 06269, United States; Philadelphia Department of Public Health, Philadelphia, PA, United States; Department of Preventive Medicine and Community Health, Morehouse School of Medicine, Atlanta, GA, United States; Sensory Evaluation Center, The Pennsylvania State University, University Park, PA 16802, United States; Department of Food Science, Pennsylvania State University, University Park, PA 16803, United States; Epidemiology, Statistics and Population Sciences, National Institute on Deafness and Other Communication Disorders, National Institutes of Health, Bethesda, MD 20892, United States; Monell Chemical Senses Center, 3500 Market Street, Philadelphia, PA, United States; National Institute on Alcohol Abuse and Alcoholism (NIAAA), National Institutes of Health, Bethesda, MD 20892, United States; National Institute on Deafness and Other Communication Disorders (NIDCD), National Institutes of Health, Bethesda, MD 20892, United States; National Smell and Taste Center, National Institutes of Health, Bethesda, MD 20892, United States; Department of Otolaryngology, University of Florida, Gainesville, FL 32610, United States; Florida Chemical Senses Institute, University of Florida, Gainesville, FL 32610, United States; National Institute on Deafness and Other Communication Disorders (NIDCD), National Institutes of Health, Bethesda, MD 20892, United States; National Smell and Taste Center, National Institutes of Health, Bethesda, MD 20892, United States; Monell Chemical Senses Center, 3500 Market Street, Philadelphia, PA, United States; Department of Computer Science, University College London, 66-72 Gower Street, London, WC1E 6EA, United Kingdom; Division of Nutritional Sciences, University of Illinois at Urbana-Champaign, Urbana, IL, United States; Department of Biomedical and Translational Sciences, Carle Illinois College of Medicine, University of Illinois Urbana-Champaign, Urbana, IL, United States; Food Science and Human Nutrition, University of Illinois at Urbana-Champaign, Urbana, IL, United States; Department of Surgery, The University of Chicago Medicine, Chicago, IL 60637, United States; Department of Otolaryngology—Head and Neck Surgery, Thomas Jefferson University, Philadelphia, PA 19107, United States; Department of Food Science and Technology, The Ohio State University, Columbus, OH 43210, United States; Department of Neurology, Massachusetts General Hospital, Harvard Medical School, Charlestown, MA 02129, United States; Monell Chemical Senses Center, 3500 Market Street, Philadelphia, PA, United States

**Keywords:** smell, taste, anosmia, hyposmia, dysgeusia, clinical

## Abstract

Millions of people in the United States experience a reduced or distorted ability to smell or taste. Chemosensory disorders such as anosmia (the inability to smell), parosmia (distorted smell), or dysgeusia (altered taste) have major impacts on health and quality of life including difficulty sensing dangers such as fire or spoilage, a diminished palatability of food and drink that can negatively influence diet and nutrition, feelings of social isolation, and an increased incidence of frailty, anxiety, and depression. Smell or taste dysfunction can also be symptoms of other health issues, including sinonasal disease, cancer, or neurodegenerative disease. Aging adults are disproportionately affected. However, smell and taste function are not regularly assessed as a part of routine healthcare despite their prevalence and impact. This is a lost opportunity, as early detection of a chemosensory disorder would enable patients to obtain needed validation, education and support for their health challenge, could direct both patient and provider to treatment options, and may suggest underlying health issues that should be addressed. To better understand the current barriers to including chemosensory testing as a regular component of health care and to identify opportunities to overcome those barriers, the conference “Towards Universal Chemosensory Testing” was convened on November 5–7, 2023, in Philadelphia, PA. This conference brought together scientists, clinicians, patients, and other experts to discuss these issues and identify collective ways to overcome barriers to testing. This white paper—which is focused primarily on the US healthcare system—is the result of those discussions.

## Introduction

Caring about health also means caring about smell and taste. The chemical senses of smell (olfaction) and taste (gustation) support our social relationships, help us find and enjoy nutritious things to eat and drink, and protect us from hazards such as gas leaks or spoiled food (Boesveldt and Parma 2020). Smell and taste disorders are common (Wysocki and Gilbert 1989). For example, the 2011–2014 National Health and Nutrition Examination Survey (NHANES)—which assessed the prevalence of chemosensory disorders in the United States at that time—found that ~18 million individuals over 40 yrs of age were experiencing smell dysfunction and ~15 million taste dysfunction (Rawal et al. 2016). The Coronavirus Disease 2019 (COVID-19) pandemic has significantly increased these numbers in the United States, with studies indicating that 77% of COVID-19 patients up to the Severe Acute Respiratory Syndrome Coronavirus 2 (SARS-CoV-2) delta variant experienced smell dysfunction and 44% experienced taste dysfunction (Hannum et al. 2020, 2023). Although chemosensory dysfunction became less prevalent with the emergence of the virus’ omicron variant (Von Bartheld and Wang 2023), millions of people continue to experience temporary or permanent smell and/or taste dysfunction resulting from COVID-19 (Sharetts et al. 2024). Indeed, chemosensory dysfunction is still a hallmark of long COVID, as indicated by the recent definition from the National Academies of Sciences, Engineering and Medicine (Fineberg et al. 2024). Indeed, the prevalence of COVID-19-associated chemosensory dysfunction, and the media attention it garnered, have increased public recognition of the importance of smell and taste far more than any public education campaign ever could.

Even so, awareness about the importance of the chemical senses to health, safety, and quality of life are often ignored or taken for granted until injury or disease compromises their function. Chemosensory loss is associated with a marked reduction in quality of life (Croy et al. 2014; Elkholi et al. 2021). Individuals with a smell or taste disorder report greater levels of depression and anxiety as compared to the general population (Kohli et al. 2016; Marin et al. 2023). These mental health consequences are in part due to the reduced pleasure associated with eating and drinking and the many social activities centered around food and drink (Nebraska Symposium on Motivation 1954). Both smell and taste, along with chemesthesis, are essential components of food flavor (Rozin 1982; Duffy and Hayes 2020). When smell or taste are lost or distorted, the food choices people make are significantly altered. These changes negatively impact nutrition and health (Reed et al. 2021). The NHANES cohort (> 40 yrs old in 2013–2014) clearly shows that smell loss is associated with elevated risk of monotonous (Rawal et al. 2021) and poor-quality diets (Rawal et al. 2016) and of related chronic diseases impacted by nutrition (e.g. obesity and cardiovascular diseases [Bhutani and McClain 2022]). This is also true for individuals with taste impairments, who have a higher prevalence of chronic conditions such as cardiovascular disease (Liu et al. 2016) and show a greater frailty score (Bernstein et al. 2021).

Chemosensory dysfunction can also be an important indicator of other diseases or disorders. For example, taste dysfunction can indicate bacterial, viral, or fungal infections of the oral cavity, malnutrition, tumors, or medication side effects (Bromley and Doty 2015; McCaughey et al. 2025). Neurodegenerative disorders including Alzheimer’s disease and Parkinson’s disease have an early olfactory component (Doty and Kamath 2014; Vohra et al. 2023). Smell loss can predate other symptoms of Parkinson’s disease by a decade or more (Haehner et al. 2011; Doty 2017), and decline in smell function predicts the progression from normal cognition to mild cognitive impairment (MCI) and from amnestic MCI to Alzheimer’s disease (Murphy 2019; Pacyna et al. 2023). Olfactory dysfunction also predicts frailty in postoperative outcomes (Mady et al. 2023), increased 5- and 10-yr mortality in older adults (Pinto et al. 2014; Devanand et al. 2015; Liu et al. 2016; Choi et al. 2021), and has been linked to adverse cardiovascular outcomes and increased risk of stroke (Chamberlin et al. 2024, 2025)

Based on a recent survey of more than 5,500 patients and caregivers, most patients reported experiencing significant difficulties having their chemosensory disorder recognized, receiving a diagnosis, or receiving adequate support (Naimi et al. 2023). This is particularly true for groups that are already disenfranchised by healthcare. Indeed, chemosensory testing is rarely available except in specialized clinical settings. The availability of universal chemosensory testing—the routine administration of direct screening chemosensory tests as part of normal health care—could significantly improve health care experiences, health outcomes, and quality of life for current and prospective patients, as well as reduce the economic burden of chemosensory dysfunction and its consequences. Increasing awareness among the public, particularly seniors and other at-risk groups, about the benefits of chemosensory testing in signposting overall health status is critical. Lessons can be learned from the promotion of hearing tests. Early detection of hearing impairment enables timely intervention with hearing aids, which can help mitigate its impact (Joint Committee on Infant Hearing 2007); for example, the well-publicized links between hearing aid use and improved cognitive health demonstrate the value of early detection and intervention in maintaining overall well-being (Yang et al. 2022; Grenier et al. 2024). Although a number of chemosensory tests have been developed and validated, many of these tests prioritize the evaluation of one sensory domain (e.g., stimulus identification, stimulus detection threshold). However, the complexity of smell or taste may not be fully captured by single-domain tests. Therefore, it is important to consider when more than one type of chemosensory test may be needed to assess diverse domains of chemosensory function (e.g. stimulus sensitivity, identification, discrimination, hedonics, preference [McCaughey et al. 2025]).

As a major step towards addressing the timely diagnosis of chemosensory disorders, 167 scientists, clinicians, and patients convened for the “Towards Universal Chemosensory Testing” (TUCT) Conference on November 5–7, 2023, at the Quorum in Philadelphia, Pennsylvania, United States, to discuss the need for increased chemosensory testing, highlight barriers to its broad implementation, and identify cross-sector priority actions to make chemosensory testing a common component of healthcare. In the first two months after the TUCT conference, a video recording of the sessions was viewed more than 1,700 times, garnered over 143,000 impressions on social media, and received over 35,000 engagements from the conference website.

TUCT was supported by a grant from the National Institute on Deafness and Other Communication Disorders (NIDCD), by attendee registration fees, and by the following conference sponsors and exhibitors: Smell and Taste Association of North America, Sensify, Inc., Berjé, Mary Bertino, Sensonics International, Burghart North America, Ahersla Health, Inc., Estenda Solutions Inc., and AROMHA, Inc. This white paper, coauthored by the TUCT conference organizers and speakers, summarizes the presentations and discussion of that event, and identifies challenges and opportunities for instituting universal chemosensory testing. Key points of emphasis included:

- Incorporating chemosensory testing as a regular part of healthcare would facilitate the diagnosis and treatment of chemosensory disorders and their numerous, associated comorbidities.
- Current chemosensory testing options are not optimized for the US healthcare system. Barriers to healthcare integration include cost, time, standardization, and the ability to adequately test all types of chemosensory disorders.
- The rarity of reimbursement for chemosensory testing by Medicare, Medicaid, or private insurance is a major barrier to incorporating this testing in primary care.
- Health and economic outcomes of chemosensory testing are not clearly established.
- Regulatory clarity is lacking on the use of chemosensory tests as diagnostic tools for both chemosensory disorders and associated diseases and disorders.
- Healthcare providers often receive minimal education and training regarding chemosensory disorders, their diagnosis and treatment, and their profound impacts on health and quality of life.
- Common data elements (CDEs) and other standardized terms and measures that could facilitate communication about chemosensory disorders in electronic health records (EHRs) and other formats are lacking.

## Chemosensory disorders

According to the NIDCD, the largest federal funder of chemosensory research in the United States (NIDCD 2025), nearly 1 in 5 Americans over the age of 40 yrs reports some alteration in their sense of taste, while nearly 1 in 4 report an alteration in their sense of smell (NIDCD 2020). This number—which has certainly risen during the COVID-19 pandemic—is four times greater than that of US adults in the same age range with uncorrectable vision (Desiato et al. 2021). Together, smell and taste disorders directly affect tens of millions of people in the United States alone. This number becomes much greater if we consider the epidemic of hidden hyposmia resulting from the COVID-19 pandemic, where people who report their smell function has recovered even though quantitative testing reveals persistent hyposmia.

Both smell and taste disorders have major impacts on the physical and mental health, safety, and well-being of those affected (Philpott and Boak 2014; Boesveldt et al. 2017; Lee et al. 2024). For example, the chemical senses serve as sentinel systems, warning of potential dangers such as fire, spoilage, and disease. Both taste and retronasal smell (i.e. input from aromas of food or drink that enter the nasal cavity via the back of the throat) are essential contributors (along with chemesthesis) to flavor. Impairment of these senses influences the food preferences and eating experience of those affected, which has a subsequent impact on people’s nutritional intake as well as their enjoyment of social interactions that occur around food or drink. Affected individuals often complain about being disconnected from others, their environment and pleasurable experiences, feelings that lead to isolation and depression (Lee et al. 2024). This isolation can be exacerbated by a lack of understanding and empathy from family, friends, and healthcare providers. Those with congenital anosmia (Karstensen and Tommerup 2012) or ageusia, who are born with a chemosensory disorder, are often undiagnosed throughout childhood. In these cases, the absence of routine chemosensory testing leaves these individuals and their parents/carers to self-diagnose. Further, while chemosensory dysfunctions may not—on their own—indicate other health problems, their diagnosis may guide the physician and patient to other tests and treatments (e.g. COVID-19, Parkinson’s disease, chronic rhinosinusitis, cardiovascular disease, cancer, depression) or lifestyle accommodations (e.g. using natural gas detectors, dating perishable foods, adjusting recipes, etc.; [Patel et al. 2022; Whitcroft et al. 2023]).

We can group chemosensory disorders into two broad categories: quantitative and qualitative conditions (Patel et al. 2022; Whitcroft et al. 2023). Hyposmia (reduced ability to smell) and anosmia (an inability to smell)—often described as “smell loss”—are the most common quantitative disorders. Qualitative disorders like parosmia (distorted smell) and phantosmia (smell phantoms) may accompany anosmia or hyposmia (sometimes referred to as microsmia). Of course, there are also equivalent quantitative and qualitative disorders in taste (e.g., ageusia for the inability to taste), though the term dysgeusia is often used to refer to both taste distortions and taste loss (Hernandez et al. 2023). While a number of objective psychophysical tests have been developed to assess quantitative chemosensory losses (Snyder et al. 2015; Doty 2019), diagnosis of qualitative disorders still largely relies on self-reports (Jafari et al. 2021; Espetvedt et al. 2023).

Chemosensory disorders can have diverse causes (Patel et al. 2022; Whitcroft et al. 2023; Lee et al. 2024). For example, burning mouth syndrome or other oral diseases affecting the salivary glands, nerve damage (such as from dental or otologic surgeries or middle ear infections), distorted tastes accompanying certain medications, viral infections (e.g. COVID-19), electrolyte abnormalities, renal insufficiency, and insult from cancer or cancer treatments (including both radiation therapy and chemotherapy) can all lead to taste loss or distortions. Similarly, there are numerous causes of olfactory disorders. These include congenital anosmia, a rare condition where individuals are born without the ability to smell, often due to a genetic disorder or abnormal development of the olfactory system (congenital ageusia is rarer still, but still reported (NIDCD 2023)). Acquired anosmia and hyposmia can occur with upper respiratory infections caused by viruses (e.g. influenza, rhinoviruses, and coronaviruses including SARS-CoV-2), bacteria, and fungi; concussion, repetitive non-concussive head trauma, or head injury; toxin exposure (e.g. pesticides); nasal inflammatory disease (e.g. chronic rhinosinusitis); severe allergies; the neurodegeneration associated with Alzheimer’s disease and Parkinson’s disease; and other causes. In many of these cases—including with neurodegenerative diseases—the advent of smell loss may be one of the earliest indicators of other serious health problems. Smell loss, or the period of recovery from it, may be accompanied by parosmia and phantosmia.

## Towards universal chemosensory testing: the conference

The TUCT conference (Table 1) included a virtual session that highlighted the perspectives of patients and other individuals affected by chemosensory disorders, six in-person sessions with multiple speaker presentations, lay summaries of the presentations led by research trainees, and an art installation inspired by olfactory loss.

**Table 1.** Chairs, speakers, affiliations, and titles of the talks at the “Towards Universal Chemosensory Testing” conference, November, 5–7, 2023.

Session 1 Patient and caregiver stories Session chairs: Katie Boateng, Pamela Silberman, Smell and Taste Association of North America		
Session 2 Chemosensory tools for population screening: The COVID-19 experience Session chair: Valentina Parma, Monell Chemical Senses Center		
*Speaker*	*Affiliation*	*Talk title*
Mark Albers, MD, PhD	Mass General Hospital/Harvard Medical School	Longitudinal smell testing to detect SARS-CoV-2 infection
John Hayes, PhD	Penn State University	The ArOMa-T, a tool for rapid, adaptive testing of olfactory thresholds
Christopher Simons, PhD	Ohio State University	Confectionary-based screening tool to assess chemosensory loss in COVID-19 patients
Pamela Dalton, PhD	Monell Chemical Senses Center	SCENTinel and other screening tools for remote testing
Session 3: **Chemosensory testing in clinical practice** Session chair: Mark Albers, Mass General Hospital/Harvard Medical School		
Katie Boateng	Smell and Taste Association of North America	The patient’s perspective
Paule Joseph, PhD	NIAAA, NIDCD	The primary care provider perspective
Jeb Justice, MD	University of Florida	The ENT perspective
Session 4: **Roadblocks to implementation of chemosensory screening** Session chair: Steven Munger, University of Virginia		
Jayant Pinto, MD	University of Chicago	It’s in the millions: epidemiological studies of chemosensory disorders
F. Abron Franklin, PhD, JD, MPH, FCPP	Philadelphia Department of Public Health	Skepticism of screening for all
Jennifer Douglas, MD	University of Pennsylvania	The insurance coverage for chemosensory testing
Edmund Pribitkin, MD, MBA	Thomas Jefferson University	The hospital administration perspective
Session 5: **Plenary addresses** Session chair: Steven Munger, University of Virginia		
Joshua Levy, MD	NIDCD	Chemosensory testing to support population screening
Richard L. Doty, PhD	University of Pennsylvania	Chemosensory testing: challenges and opportunities
Session 6: **Success stories of population screening of sensory function** Session chair: Kai Zhao, Ohio State University		
Howard Hoffman, PhD	NIDCD	Population screening of sensory function
Susan Coldwell, PhD	University of Washington	NIH Toolbox: what did we learn?
Valerie Duffy, PhD	University of Connecticut	The NHANES experience: chemosensory testing in large cohort
Julie A. Mennella, PhD	Monell Chemical Senses Center	Chemosensory phenotyping in children: lessons learned and challenges ahead
Yanina Pepino, PhD	University of Illinois at Urbana-Champaign	
Session 7: **Imagining a chemosensory-screened world** Session chair: Mark Albers, Mass General Hospital/Harvard Medical School		
Patrice Hubert, PhD	Monell Chemical Senses Center	Supporting long-term consequences of chemosensory loss: nutrition and mental health
Duncan Boak	Fifth Sense	Piloting smell testing tools in the home, workplace, and public health settings in the UK
Linda Barlow, PhD	University of Colorado Anschutz Medical Campus	Taste testing: impact on basic science and cancer care
Marianna Obrist, PhD	University College London	Digital smell technology: the future with routine chemosensory testing

### Session 1: Patient and caregiver stories—listening session

During the 2-h virtual event opening the conference, four individuals living with olfactory dysfunction shared their stories live. This live panel was complemented by a series of short videos and chat comments (available at https://www.kudoboard.com/boards/Tem2GHnT) provided by patients, scientists, clinicians, and advocates that provided insightful accounts of the impact of chemosensory disorders on a person living with olfactory or taste dysfunction, including on nutrition, cooking, relationships, hygiene, mental health, and safety. Further, there was a consistent theme of the importance of chemosensory testing to validate one’s experience of loss and medically document their dysfunction. Many shared that smell and taste testing could have changed the course of their lives. Each patient described their path to finding support and medical care as a painful, long, and isolating process. Scientists and clinicians have shared how knowing who has a chemosensory disorder will help direct their research. Indeed, clinicians and researchers are still struggling to identify the prevalence of chemosensory dysfunction, particularly after COVID-19, limiting research on treatment strategies. Clinicians also shared how their practice would change if routine measures of smell and taste allowed them to improve the diagnosis and treatment of disease, which expanded beyond chemosensory disorders into neurodegenerative, neurological, and mental health issues, among others.

### Session 2: Chemosensory tools for population screening: the COVID-19 experience

The first live session of the conference reported results from four studies supported by the National Institutes of Health’s Rapid Acceleration of Diagnostics (RADx) program, which was launched in the Summer of 2020 to accelerate approaches to diagnosing and responding to COVID-19. The RADx-radical (RADx-rad) projects (Adams 2024) were particularly focused on out-of-the-box approaches and included efforts to develop chemosensory testing strategies that were objective, self-administered, and easily deployable and that could be used in screening and disease surveillance efforts to identify potential COVID-19 disease (a number of established chemosensory tests currently used in some clinics and/or research studies were discussed in a later keynote address and in other sessions, see below). While the greater availability of SARS-Cov-2 testing lessened the need for tests more focused on COVID-19 symptoms, the development of new chemosensory tests under this mechanism could prove valuable for overcoming barriers of cost, time, and accessibility when assessing chemosensory functions. Members of four RADx-rad teams described studies with four chemosensory tests that were at different stages of research and clinical validation. Three card-based tests use peel-and-burst technologies to release odorants and can all be self-administered. AROMHA is a 5-min. at-home smell test that includes odor intensity rating, odor percept identification, and odor discrimination, memory, and intensity assessment (Jobin et al. 2024). SCENTinel is a < 2-min. smell test that measures odor detection, intensity, identification, and pleasantness (Parma et al. 2021; Hunter et al. 2023, 2024, 2024). The Adaptive Olfactory Measure of Threshold (ArOMa-T) pairs an odorant delivery card with a web app employing an adaptive Bayesian algorithm to rapidly determine an odor detection threshold (Weir et al. 2022). Finally, a candy-based test can assess retronasal smell (an essential component of flavor) as well as sweet and sour taste perception (Man et al. 2022).

### Session 3: Chemosensory testing in clinical practice

In this session, a patient, a nurse scientist/nurse practitioner, and a rhinologist shared first-hand experiences with the diagnosis and treatment of chemosensory disorders. Modern clinical practice includes multidisciplinary teams and tight schedules for interactions with patients. This reality presents significant challenges in the diagnosis and management of chemosensory disorders, which require careful assessment, patient counseling, and individualized treatment plans. One major issue highlighted was the difficulty patients face in getting access to a knowledgeable and caring provider (Ball et al. 2021), as well as challenges for finding sufficient time for specialist appointments and a high economic cost to the patient. The cornerstone of preventive and primary care is the ability to detect, diagnose, and manage conditions early to improve patient outcomes. However, chemosensory testing and screening have been challenging due to the lack of standardized, efficient, and accessible testing methods that can be easily integrated into clinical practice. Another significant challenge discussed was the inadequate time available (sometimes 15–30 mins per patient) under typical health care system financial models to conduct thorough diagnostic evaluations and provide appropriate counseling to patients (van Baal et al. 2018). Furthermore, the session emphasized that health insurance coverage for smell and taste testing is insufficient (Saraswathula et al. 2023), with many insurance plans either not covering chemosensory testing or offering only partial reimbursement (also covered in session 4). This lack of coverage limits early and accurate diagnosis and restricts treatment options, increasing the patient’s frustration and suffering.

### Session 4: Roadblocks to implementation of chemosensory testing

During this session, a diverse panel of clinicians, hospital administrators, and public health officials discussed the current roadblocks to implementing chemosensory testing in health care systems and in the public health arena. One major challenge is the lack of standardization, as there is no universally adopted method of testing in clinical practice. Secondly, it was noted that the costs of chemosensory testing, including the costs of the tests themselves, are rarely reimbursed by health insurance (Saraswathula et al. 2023). Low reimbursement rates compared to other tests and procedures of similar complexity and duration are also a disincentive to the embrace of chemosensory testing by both providers and health care systems. The absence of sufficient diagnostic and procedure codes for chemosensory disorders and testing, critical for billing, contributes to this problem. It was also noted that it is tremendously valuable to include chemosensory testing in major epidemiological and population health studies. Partnerships with industry and community stakeholders that could be impacted by chemosensory disorders were suggested as a fruitful avenue for facilitating chemosensory testing in public health.

### Session 5: Keynote lectures

Two speakers presented broad views of the state of chemosensory testing. Joshua Levy, MD, Clinical Director for the NIDCD—which supports research on normal and disordered smell and taste—discussed new efforts at the NIDCD to enhance the health and quality of life of individuals affected by chemosensory disorders. Of particular note was the announcement of a new National Smell and Taste Center, based in the NIDCD, that will work to enhance research, comprehensive care, education, and outreach for the chemical senses (2024).

Richard L. Doty, PhD, founding director of the University of Pennsylvania Taste and Smell Center, presented a historical perspective of chemosensory testing. He included numerous examples of how modern testing tools, including well-validated, self-administered tests as the 40-item University of Pennsylvania Smell Identification Test (UPSIT, [Doty et al. 1984]) and its 12-item variant, the Brief Smell Identification Test (B-SIT or CC-SIT, (Doty et al. 1996]), have led to important understanding of the prevalence and impact of chemosensory disorders on human health. For example, it was noted that olfactory testing contributed to the recognition that olfactory dysfunction is often one of the earliest signs of neurodegenerative disease (Doty 2017).

### Session 6: Success stories of population screening of chemosensory function

Our knowledge of the prevalence of chemosensory disorders comes largely from several efforts to develop and implement validated rapid testing strategies that can be more easily deployed in large populations. Smell and taste tests developed for the NIH Toolbox initiative (Coldwell et al. 2013; Dalton et al. 2013) were an effort to establish a common standard for chemosensory assessments across the lifespan (from 3 yrs old) that could be administered rapidly and as part of larger health screenings.

The 2011–2014 version of the NHANES built on the work of the NIH Toolbox initiative to deploy modified versions of these smell and taste tests that would fit within the strict limits (e.g. time to administer) of the NHANES protocol (Hoffman et al. 2016). Challenges to large scale population screening, including the short time windows to incorporate chemosensory tests amongst the many other assessments in these large studies, and the distinct testing needs of participants of different age groups, life experiences, and cognitive abilities, were discussed. Importantly, testing in different syndromic populations highlights the need to assess multiple chemosensory modalities and multiple domains within a sense (e.g. stimulus identity, stimulus detection threshold, preference, etc), as not all may be similarly impacted in a particular patient or particular condition/disease state (Kulaga et al. 2004; McEwen et al. 2007; Alfaro et al. 2020).

### Session 7: Imagining a chemosensory-screened world

There are many ongoing efforts to increase the prevalence of chemosensory screening. In the United Kingdom, partnerships between the smell and taste disorders charity Fifth Sense and the UK’s largest natural gas distribution network, Cadent, have led to an educational program and engagement with professional and public audiences to increase awareness of the safety implications associated with olfactory dysfunction. The partnership has also resulted in the joint development of a “scratch and sniff” gas safety leaflet that enables homeowners to test their own ability to detect mercaptan, the odorant added to odorless natural gas to warn people of gas leaks. This is now being used by the UK Fire and Rescue Service as part of safety visits to the homes of members of the public in some parts of the United Kingdom. Fifth Sense also works with other commercial partners as well as with otolaryngologists and other healthcare providers around the United Kingdom to promote awareness and assessment of chemosensory disorders and chemosensory testing. The speakers noted that the persistent effects of olfactory and taste disorders have long-term negative consequences for nutritional and mental health, which are often unaddressed. While olfactory testing gets most of the attention, speakers emphasized that taste disorders are a significant health issue, as well, particularly for cancer patients undergoing chemotherapy or targeted radiation therapy.

Finally, it was observed that emerging digital technologies could enhance the accuracy, usability, and patient acceptability of chemosensory tests (Hopper et al. 2024). Such technological advances offer the possibility that individuals can one day self-administer smell tests in a range of settings, including at home, allowing smell healthcare services to evolve and become part of a routine practice and self-care culture that could span smell, taste, and eating (Vi et al. 2024).

### Other presentations: lay summaries and olfactory art

A major goal of the conference was to promote access to its content. Research trainees provided lay summaries of material presented by the speakers and highlighted their research. Key content was translated into Spanish and posted on social media. Social media channels were also used to publicize the conference and to communicate highlights. Finally, conference participants had the opportunity to engage with an interactive art piece created by an artist with congenital anosmia, offering a compelling visual and sensory representation of their experience with an olfactory disorder (https://itp.nyu.edu/thesis2023/projects/?student-id=9).

## The current state of chemosensory testing

### Assessing chemosensory function

Multiple aspects of chemosensory function can be measured, including stimulus identification (e.g. rose vs. lemon, or sweet vs. bitter), discrimination (e.g. “that smells different than the other two”), and detection threshold (i.e. the lowest stimulus concentration that can be reliably detected), some of which can be measured from an early age (McCaughey et al. 2025). Odor or taste memory, supra-threshold intensity, or hedonic attributes can also be assessed. Most modern clinical chemosensory tests often assess just one or some of these measures using established psychophysical methodologies and with stimuli presented via the nostrils (for smell) or in the mouth (for taste). Performance on most of these tests, at least for smell, is highly correlated and has been found to load on one common factor in principal components analysis (Doty et al. 1994; Boucai et al. 2011); that is, different measures are assumed to share the same underlying variance in performance across individuals and thus are nominally redundant in clinical usage (note that some studies suggest this is not quite true, [Koskinen et al. 2004; Alfaro et al. 2020]). The field of chemosensation phenotyping remains in its nascent stages relative to hearing or vision assessments, particularly regarding population screening methodologies. The optimization of protocols to maximize data acquisition efficiency while maintaining methodological rigor is ongoing, as researchers continue to elucidate both normative developmental trajectories and pathological manifestations.

Stimulus delivery is a key component of any chemosensory test. Odorants and tastants must be consistently presented to maintain stimulus quality (i.e. what the chemical is) and its concentration. Several different delivery systems have been employed over the years. In sophisticated research settings, olfactometers or gustometers can be used. However, in more typical settings, including the clinic, olfactory tests often rely on encapsulated odors (e.g. scratch-and-sniff or peel-and-burst labels) or odor-impregnated pens as an odor source (Doty 2019). Taste tests often rely on embedded matrices that can be placed in the mouth (Snyder et al. 2015; Doty et al. 2021).

Chemosensory tests can vary in the cost of the test itself, the time it takes to complete the test, and whether the test can be self-administered or must be administered by a trained individual. Each of these factors can influence whether a test is appropriate or even feasible for a particular clinic, public health, or research setting.

While many existing tests have proven quite effective for diagnosing chemosensory disorders, each has its limitations. For example, identification tests partially conflate sensory performance with naming ability, memory, and familiarity (Pangborn et al. 1988; Chrea et al. 2004). These factors, and perception more generally, vary with cognitive status and culture. By contrast, tests of detection threshold, which are generally less reliable (Doty et al. 1995), can take significantly longer than those for odor identification, and often require a trained individual to administer them, adding both time and labor costs. Some tests are inappropriate for different individuals and groups (including young children) due to factors such as language barriers, cognitive impairment, or experience with testing. Those with cognitive challenges are expected to grow as the population ages, making this issue increasingly relevant when assessing older adults. This is particularly significant given the shifting demographics of aging, especially in developed countries. The prevalence of chemosensory dysfunctions associated with COVID-19, along with specific funding from the NIH, have spurred efforts to develop new chemosensory tests that can mitigate some of these limitations, complement the strengths of established tests, and fill specific screening and testing niches. For example, tests designed for rapidly screening populations in schools, workplaces, or other community settings may emphasize speed, self-administration, and low cost but lack the ability to comprehensively assess the breadth and degree of chemosensory deficits. By contrast, more extensive tests that assess more stimuli and/or several chemosensory domains (e.g. identification, detection threshold, discrimination) can provide health care providers with a more detailed understanding of a patient’s chemosensory dysfunction, although they typically require more expertise and resources to administer and analyze.

#### Barrier 1

Chemosensory tests can be cost-prohibitive in many health care settings where costs of the tests themselves are not reimbursed by insurance or cannot be absorbed by the health care provider.

#### Opportunity 1

The development of new, lower cost tests may increase the willingness of insurance providers to cover test costs or alternatively minimize the cost burden on providers or patients.

#### Barrier 2

Modern clinical visits in the United States are typically short in duration. There is limited time for patient and provider to discuss health concerns or test results, let alone to devote provider effort to administering additional tests. Indeed, the time needed to take the most common chemosensory tests can also be incompatible with the workflow of a typical clinic session. These time and personnel effort constraints provide a disincentive to include chemosensory testing as a part of normal healthcare interactions.

#### Opportunity 2

A multi-stage testing regimen could mitigate some of these challenges. Validated self-administered chemosensory tests could be taken at home prior to a clinical visit, with test responses entered into EHR systems automatically or by the patient for review by the provider. This would eliminate the need for a test administrator and would allow providers to focus on test results, not test taking. Test responses indicating a chemosensory dysfunction or a change in chemosensory responses compared to prior tests could suggest a referral to a specialist (e.g. otolaryngologist, neurologist) or a testing center for more comprehensive assessments and/or diagnosis.

#### Barrier 3

The spectrum of chemosensory tests currently used in US clinics vary in their methodology, scoring, and normalization. This lack of standardization creates challenges not only with interpretation by non-specialist providers, and sharing health information between providers, but can also be a barrier in data management, including entering test results into EHR systems.

#### Opportunity 3

Creating common workflows and results management pipelines to easily incorporate chemosensory test results into EHRs, compare those results to normative data or to previous test results, indicate chemosensory function levels (e.g. normosmia, hyposmia, anosmia) and suggest likely etiologies based on algorithms incorporating the chemosensory test result with other data in the EHR, would facilitate chemosensory test use by non-specialists and by health systems.

#### Barrier 4

While there are a variety of chemosensory tests adept at identifying quantitative chemosensory disorders, there is a paucity of tools to objectively assess the presence of a qualitative chemosensory disorder (e.g. distortions or phantoms).

#### Opportunity 4

While recent studies suggest that some existing chemosensory tests may be useful in detecting parosmia (Liu et al. 2020; Hunter et al. 2023), it is imperative to develop new tests optimized for parosmia and/or phantosmia.

### Insurance reimbursement for chemosensory testing

For smell or taste testing to be affordable in most contexts, the costs of the tests themselves, as well as provider time and resources needed to administer and interpret these tests, must be recoverable. However, despite the prevalence of chemosensory disorders, their impacts on patient health and quality of life, and their relevance as indicators for various diseases and disorders, chemosensory testing is not regularly reimbursed by health insurers (Saraswathula et al. 2023; Independence Blue Cross 2025).

#### Barrier 5

Insurers typically require several standardized codes that describe what care a patient received and why a particular procedure was performed before they will reimburse the patient or provider for the costs of medical services or procedures. International Classification of Diseases-10th edition (ICD-10) codes, which are maintained by the World Health Organization (WHO), represent specific diagnoses and health conditions. A few relevant ICD-10 codes do exist for chemosensory disorders such as anosmia and parosmia (Table 2, ICD-10-CM 2025). However, several chemosensory disorders (e.g. phantosmia, hyposmia, ageusia) do not have their own unique code and instead must be represented by catch-all codes such as R43.8 (Other disturbances of smell and taste). Creating specific ICD-10 codes for each chemosensory disorder would likely simplify insurers’ assessments of which interventions are eligible for reimbursement in conjunction with a specific diagnosis.

**Table 2.** Procedure and billing codes in the US healthcare system.

Code Type	Code	Description
International Classification of Disease-version 10 (ICD-10) codes		
**R43 Disturbances of smell and taste**		
	R43.0	Anosmia
	R43.1	Parosmia
	R43.2	Parageusia
	R43.8	Other disturbances of smell and taste
	R43.9	Unspecified disturbances of smell and taste
**Current Protocol Terminology® (CPT®) codes**		
	92700	Other otorhinolaryngological services or procedures
	41599	Other procedures on the tongue and floor of mouth
**Healthcare Common Procedure Coding System (HCPCS) Level II codes**		
No Level II codes were identified for smell or taste testing supplies		

* The 92700 and 41599 codes, while indicated by some insurers as appropriate for reimbursement of procedures related to smell or taste disorders, have in practice shown a poor track record of facilitating cost reimbursement related to chemosensory testing.

By contrast, Current Procedural Terminology® (CPT®) codes are primarily used to identify specific procedures and other medical services, particularly for insurance billing purposes. CPT codes are maintained and updated annually by the American Medical Association (AMA). Medicare and Medicaid use the related Healthcare Common Procedure Coding System (HCPCS) Level I codes, maintained by the US Center for Medicare and Medicaid Services (CMS) and based on the CPT® code system, for this equivalent purpose. However, no specific CPT® or HCPCS Level I codes exist for either smell or taste testing. Some providers may use the CPT code 92700 for otolaryngological services that do not have their own code or CPT® 41599 for procedures performed on the tongue or floor of the mouth (Saraswathula et al. 2023). However, any payment on these codes (if made) may not sufficiently reimburse testing costs or take into account cost differences associated with specific tests. Thus, specific CPT® codes (and, by extension, HCPCS Level I codes) are needed for chemosensory testing.

#### Opportunity 5

The detailed processes needed to establish new codes are complex (e.g. American Medical Association (2025)) and beyond the scope of this manuscript. However, we will highlight some key strategies to realize this goal. Most importantly, there should be coordinated efforts between relevant professional organizations (e.g. the American Academy of Otolaryngology—Head and Neck Surgery, American Academy of Neurology) and patient advocate groups. Such organizational partnerships could lobby the WHO to create specific ICD-10 codes for each chemosensory disorder or advance submissions to the CPT® Editorial Panel of the AMA to establish specific CPT® codes for the use of chemosensory tests as a part of regular health screening or in the diagnosis or assessment of chemosensory disorders or comorbidities such as neurodegenerative disease.

These discussions between patient groups and professional organizations could include defining evidence required for reimbursement, details which could be incorporated into payment requests. This is a necessary step before private health insurers and Medicare and Medicaid will regularly provide reimbursement for testing. In the meantime, some private insurers could support reimbursement for the chemosensory testing devices themselves through HCPCS Level II codes, likely within the A (medical or surgical supply) or S (temporary) categories. Unfortunately, to the best of our knowledge, no HCPCS Level II code currently exists for smell or taste testing. Further, Medicare and Medicaid do not support the S code category, making the use of this code category an imperfect solution at best.

#### Barrier 6

The limited treatment options (Patel et al. 2022; Whitcroft et al. 2023) for chemosensory disorders raises the question of the value of smell testing for the patient and for the insurer. How will measuring chemosensory function impact the patient’s health as well as future clinical care decisions? Will diagnosing a chemosensory disorder through regular testing save money down the road?

#### Opportunity 6

Rigorous studies are needed to determine the economic and health outcome value of chemosensory testing and to justify reimbursement by insurance providers (as well as to encourage greater efforts towards developing new therapies). At this time, such studies do not exist. However, we can posit some likely outcomes. Patients with symptomatic chemosensory disorders have increased incidence of depression and anxiety (Kohli et al. 2016; Kamath et al. 2018), increased frailty (Bernstein et al. 2021; Mady et al. 2023), and decreased lifespan (Pinto et al. 2014; Devanand et al. 2015; Choi et al. 2021). The diagnosis of chemosensory disorders would provide these patients with validation of their perceived disability, increase referrals for mental health care, peer support, nutrition and dietary counseling, and improve their quality of life. Chemosensory dysfunction can be a sentinel sign or symptom of other diseases, such as cancer, sinonasal disease, Parkinson’s, and Alzheimer’s disease, skull base cerebrospinal fluid leaks, or multiple sclerosis. The diagnosis of occult or manifest chemosensory disorders would expedite diagnoses of these comorbidities and lead to early intervention and thus more efficacious and cost-effective management of debilitating disease.

#### Barrier 7

Chemosensory testing is not an end unto itself. Test results should guide the patient and their health care provider to next steps, whether those include immediate treatment or referral to an appropriate specialist (e.g. an otolaryngologist for additional testing and treatment related to sinonasal issues, a neurologist if neurological disease is suspected, dieticians or nutritionists to support healthier eating, a psychologist or psychiatrist if depression or anxiety is an issue). While anecdote suggests all of these are common paths for those who are diagnosed with a chemosensory disorder, data indicating which of these are most often engaged and what impacts each has on patient outcomes and overall healthcare costs remains unclear.

#### Opportunity 7

Studies are needed that assess patient outcomes such as improvement in chemosensory function and quality of life, diagnosis, treatment of comorbidities identified as a result of chemosensory testing, and psychosocial support. The acceptability of diagnostic and treatment options to patients must also be determined.

#### *Barrier* 8

Olfactory tests used to determine the presence of an olfactory disorder are considered Class II devices by the US Food and Drug Administration (FDA) and are 510(K) exempt from premarket notification requirements (FDA 2025). This FDA guidance has facilitated the development and use of various olfactory tests in the diagnosis of anosmia and hyposmia. However, this same guidance states “when indicated for the screening or diagnosis of diseases conditions other than the loss of olfactory function, the (olfactory test) is not exempt.” This determination adds substantial requirements for using objective measures of olfactory function in the diagnosis of COVID-19, various neurodegenerative diseases, or other diseases or disorders where hyposmia or anosmia may serve as a useful biomarker. Taste tests, including electrogustometers, are considered as Class I medical devices by the FDA and are exempt from premarket notification (Center for Devices and Radiological Health 2024). Home screening tests for chemosensory disorders may offer an opportunity for early detection that could help individuals monitor their health. While not diagnostic, such tests can encourage timely intervention, reducing long-term healthcare costs by prompting users to seek professional care when necessary or aware of dysfunction. There may also be an opportunity to leverage home screening data in clinical trials or research initiatives, providing valuable insights into early disease progression and supporting diagnostic efforts in partnership with healthcare providers. Although such tests may not serve as diagnostic tools, they still face scrutiny in terms of reliability and acceptance by medical providers, as well as concerns around reimbursement.

#### *Opportunity* 8

Industry has numerous opportunities to innovate chemosensory testing, including EHR integration and expansion of diagnostic claims. Wider adoption of smell and/or taste as a vital sign or critical biomarker embedded into clinical trials requires educating industry and tech-focused venture groups to provide incentives for industry investment. With evidence from these trials, both smell and taste tests could obtain full approvals for use as screening or diagnostic tools or as companion diagnostics for the diseases under investigation in these clinical trials. For instance, a smell test is embedded in a clinical trial of an anti-inflammatory drug for Alzheimer’s disease and amyotrophic lateral sclerosis (NCT 05189106). However, obtaining full or companion FDA approvals is costly, and it may require the coordinated effort of multiple industry stakeholders to lead (and finance) this process.

### Provider knowledge and education

Little time is devoted to educating future health professionals (physicians, nurses, advanced practice providers, dentists, dieticians, etc.) about the chemical senses or their associated disorders. Indeed, some professional school curricula may provide no formal coverage of these topics beyond a brief introduction if the student participates in specialized training such as in otorhinolaryngology clerkships. This lack of healthcare provider awareness is witnessed by patients, who often feel providers are unaware of chemosensory disorders, the impact these disorders have on health or quality of life, or the testing or treatment options that are currently available.

#### Barrier 9

The lack of attention to the chemical senses in healthcare education reflects, at least in part, that accreditation exams—such as the United States Medical Licensing Examination (medicine), the National Council Licensure Examination (nursing) and the Integrated National Board Dental Examination (dental)—do not emphasize content related to chemosensory disorders. As curriculum time is limited, information and experiences that are not prioritized on accreditation exams see reduced attention in the classroom and during clinical experiences, and trainees are not incentivized to study or review this material.

#### Opportunity 9

Coordinated advocacy by patient groups and professional societies could change this, creating downward pressure on educational institutions to increase educational content related to anosmia, dysgeusia, and other chemosensory disorders. Advocacy efforts should be focused on accreditation organizations and on institutional associations—such as the Association of American Medical Colleges, the American Association of Colleges of Nursing, and the Commission on Dental Accreditation, and the American Dental Education Association—that help set national priorities for health care professional education. For example, in the United Kingdom, the efforts of Fifth Sense and Professor Carl Philpott have resulted in anosmia being added to the list of presenting symptoms on the General Medical Council’s Medical Licensing Assessment content map (Fifth Sense 2022). This means that all medical graduates in the United Kingdom will need to understand this chemosensory impairment and how to consider a differential diagnosis for it. In the United States, the National League for Nursing has started a Strategic Action Group to integrate chemosensory science into undergraduate and graduate nursing curricula (private communication).

#### Barrier 10

Only specialists (e.g. rhinologists, dentists specializing in oral medicine) have regular professional opportunities to enhance their knowledge and expertise in the diagnosis and treatment of smell or taste disorders. By contrast, a general practitioner is less likely to prioritize professional education about the advantages and disadvantages of the many different types of chemosensory tests, how potential treatment options for a specific disorder (e.g. anosmia) may differ by etiology, or what psychosocial challenges chemosensory disorder patients may be experiencing. This is problematic given the prevalence of manifest and occult chemosensory disorders, as a general practitioner is typically the first point of contact for a patient newly experiencing olfactory or taste dysfunction. This provider is likely seeing one or more undiagnosed patient each day, making it even more difficult for that patient to be diagnosed and receive care.

#### Opportunity 10

Post-graduate and continuing education requirements for healthcare professionals provide opportunities to introduce general practitioners to chemosensory disorders, their impact on patients, and current best practices for diagnosis, treatment, and specialist referral. Professional organizations that create curricula for continuing education, such as the American Academy of Family Physicians, could design modules focused on chemosensory disorders and testing that would provide consistent, validated, and up-to-date information for their members.

### Data sharing to enhance diagnosis and treatment

If chemosensory testing is to be integrated into clinical care, there must be standardization of terminologies, testing methodologies, and the interpretation of results. This does not mean that a single type of measurement (e.g. identification vs. detection threshold) should be exclusively employed. Indeed, testing different aspects of chemosensory function may be advantageous in many cases. Rather, there should be an expert consensus on what type of test should be used under which circumstances. For example, screenings of large groups (e.g. screening schoolchildren for congenital anosmia) might best be accomplished by a self-administered rapid detection threshold or discrimination test (odor identification may be problematic in children with limited lived experience or diverse cultural backgrounds). Yearly testing by a general practitioner should combine low cost and self-administration with the ability to quantify changes over time. Specialist testing could be more involved and include multiple domains (e.g. threshold, discrimination, identification, memory).

#### Barrier 11

Terminology is not always standardized. For example, the term dysosmia is more typically used as an umbrella term for qualitative olfactory disorders (e.g. parosmia, phantosmia), but less commonly to encompass both quantitative (anosmia and hyposmia) and qualitative olfactory disorders (Hernandez et al. 2023). Smell tests can also be referred to in different ways. For example, the University of Pennsylvania Smell Identification Test is often abbreviated in the literature as “UPSIT” but sometimes is referred to as “SIT-40” (to indicate that it has 40 questions). The absence of standardized terminology, particularly in EHRs and in clinical trials, can create confusion and undermine effective clinical care and research.

#### Opportunity 11

Terminology should be standardized by establishing CDEs that are used throughout clinical research and EHRs. Professional organizations (e.g. the American Academy of Otolaryngology, the Association for Chemoreception Sciences) should work with the National Institutes of Health, the Center for Medicare and Medicaid Services, and the AMA to establish CDEs and other standardized nomenclature to ensure clarity of communication and interpretation in both research and clinical care. To further support this effort, a National Academy of Sciences, Engineering, and Medicine (NASEM) consensus study could comprehensively evaluate the evidence for smell and taste testing. This independent, evidence-based review would provide authoritative guidance on integrating olfactory assessments into health policy, clinical standards, and scientific initiatives.

#### Barrier 12

If researchers are to advance the science of diagnostics and treatments, and clinicians are to learn best practices, data must be maintained in ways that are both secure (protecting patient and provider privacy) and accessible. Further, accessibility must not only include making data available but also ensuring that data is maintained in formats that all stakeholders can obtain and analyze.

#### Opportunity 12

The US Department of Health and Human Services, or one of its member agencies, should establish one or more databases to house data related to chemosensory testing. Obligations to deposit data in these databases could be linked to the receipt of federal research funds, and formats and expectations for minimal information should be delineated. PubMed Central (Pinto et al. 2014), a database of research manuscripts that report NIH-funded studies, and MINSEQE (FGED 2025), a database for Next-Gen sequencing, are two useful models.

## Prioritizing next steps

High-quality chemosensory testing options have been available for years. However, the implementation of chemosensory testing as a normal part of health care has lagged far behind similar testing for other sensory functions, such as hearing or vision. The minimal presence of chemosensory testing in modern health is due to multiple factors, which include costs that are not typically reimbursed by health insurance, testing formats that may not be optimized for different types of clinical environments, minimal education of most health care providers (and patients, as well) on the subject, and poorly defined outcomes expectations and cost-benefit analyses.

Above, we detail these barriers and more and suggest opportunities to overcome them. But which actions should be prioritized? In our view, the first priority (and one that the TUCT conference was designed to facilitate) is coordinated action amongst key stakeholders—including professional organizations, researchers, educators, patient groups, and industry leaders—to develop and resource a clear, strategic roadmap. This effort should focus on three critical goals within the next five years:

- Standardization: Establishing CDEs and standardized nomenclature for smell and taste testing to ensure consistency in research and clinical application.
- Regulatory and Reimbursement Pathways: Developing the necessary infrastructure to support widespread implementation, including the creation of relevant diagnostic and procedure billing codes, securing FDA approvals for validated tests, and advocating for insurance reimbursement.
- Education and Public Awareness: Embedding chemosensory assessment into medical and health sciences curricula while driving public health campaigns to raise awareness of olfactory and taste disorders as critical health indicators.

Ultimately, driving meaningful change requires a unified commitment to research, policy development, and public engagement—one that this group, alongside a broader cross-sector community, is prepared to lead.
